# Simultaneous two-photon activation of presynaptic cells and calcium imaging in postsynaptic dendritic spines

**DOI:** 10.1186/2042-1001-1-2

**Published:** 2011-01-26

**Authors:** Masanori Matsuzaki, Graham CR Ellis-Davies, Yuya Kanemoto, Haruo Kasai

**Affiliations:** 1 Laboratory of Structural Physiology, Center for Disease Biology and Integrative Medicine, Graduate School of Medicine, University of Tokyo, Tokyo, Japan; 2 Center for NanoBio Integration, University of Tokyo, Tokyo, Japan; 3 PRESTO, CREST, Japan Science and Technology Agency, Saitama, Japan; 4 Current Address: Division of Brain Circuits, National Institute for Basic Biology, Okazaki, Japan; 5 Department of Neuroscience, Mount Sinai School of Medicine, New York, NY 10029, USA

## Abstract

**Background:**

Dendritic spines of pyramidal neurons are distributed along the complicated structure of the dendritic branches and possess a variety of morphologies associated with synaptic strength. The location and structure of dendritic spines determine the extent of synaptic input integration in the postsynaptic neuron. However, how spine location or size relates to the position of innervating presynaptic cells is not yet known. This report describes a new method that represents a first step toward addressing this issue.

**Results:**

The technique combines two-photon uncaging of glutamate over a broad area (~500 × 250 × 100 μm) with two-photon calcium imaging in a narrow region (~50 × 10 × 1 μm). The former was used for systematic activation of layer 2/3 pyramidal cells in the rat motor cortex, while the latter was used to detect the dendritic spines of layer 5 pyramidal cells that were innervated by some of the photoactivated cells. This technique allowed identification of various sizes of innervated spine located <140 μm laterally from the postsynaptic soma. Spines distal to their parent soma were preferentially innervated by cells on the ipsilateral side. No cluster of neurons innervating the same dendritic branch was detected.

**Conclusions:**

This new method will be a powerful tool for clarifying the microarchitecture of synaptic connections, including the positional and structural characteristics of dendritic spines along the dendrites.

## Background

The microarchitecture of synaptic connections determines information processing in cortical circuits. The inter/intra-layer and inter/intra-columnar architecture of synaptic connectivity has been revealed by laser-scanning one- or two-photon stimulation of neurons with caged glutamate [1-7]. Although previous experiments have measured the amplitude of postsynaptic currents or depolarization, they have not been able to identify the sites of synaptic connections.

The structure and location of dendritic spines, the major postsynaptic sites of excitatory synapses, are crucial to information integration in the postsynaptic cell [8]. Spine size correlates well with the number of functional α-amino-3-hydroxy-5-methyl-4-isoxazolepropionic (AMPA) receptors [9-11]. Dendritic spine location determines the extent to which depolarization spreads into the soma, the local dendritic spike, and synaptic plasticity [12,13]. Induction of nonlinear depolarization had been suggested to require the activation of dozens of dendritic spines along the same dendritic tree within a narrow time window (~6 ms) [14,15]. Functionally or spatially associated pyramidal cells innervating the same dendritic branch of a postsynaptic cell may cause correlated activation in the brain. In order to examine this possibility, the location of presynaptic cells and the size and location of the dendritic spines that they innervate must be determined.

Although it is possible to determine the structure and location of synaptic connectivity by staining pair-recorded cells, it is very difficult to identify pairs of connecting cells over a relatively broad area and then find their synaptic sites [16-19]. In this study, we developed a new method that combines calcium (Ca^2+^) imaging with photostimulation via two-photon macro photolysis of caged glutamate (2pMAPG) [7]. The volume of uncaged glutamate is restricted to the focal volume of the laser beam. Thus, it is possible to map the approximate positions of photostimulated cells that induce postsynaptic currents in a patch-clamped cell via three-dimensional (3D) scanning of laser focal volumes from 2pMAPG in slices of rat cortex [7]. During 2pMAPG mapping in layer 2/3, we also performed Ca^2+ ^imaging in the dendrites of layer 5 pyramidal cells and identified dendritic spines in which photostimulated cells triggered 2pMAPG-mediated Ca^2+ ^transients. We found that spines located distal to their parent soma were innervated preferentially by cells on the ipsilateral side.

## Results

### Activation of layer 2/3 pyramidal cells with 2pMAPG mapping

First, the 3D 2pMAPG mapping of induced action potentials (APs) was validated in layer 2/3 pyramidal cells in the rat motor cortex. In order to activate a large number of glutamate receptors to induce APs, the focal volume for 2pMAPG was expanded with a 720-nm laser beam [7]. The point-spread function of the focal volume for 2pMAPG was estimated using 0.1-μm fluorescent beads at lateral and axial full-width at half-maximum values (FWHMs) of 1.03 ± 0.04 μm and 15.4 ± 0.7 μm (*n *= 11), respectively. These lateral and axial FWHMs were 3.8- and 9.8-fold longer, respectively, than those for two-photon imaging using the 830-nm laser beam (see Methods). Therefore, the 2pMAPG volume was approximately 140-fold larger (3.8 × 3.8 × 9.8) than that obtained by two-photon imaging.

**Figure 1:**
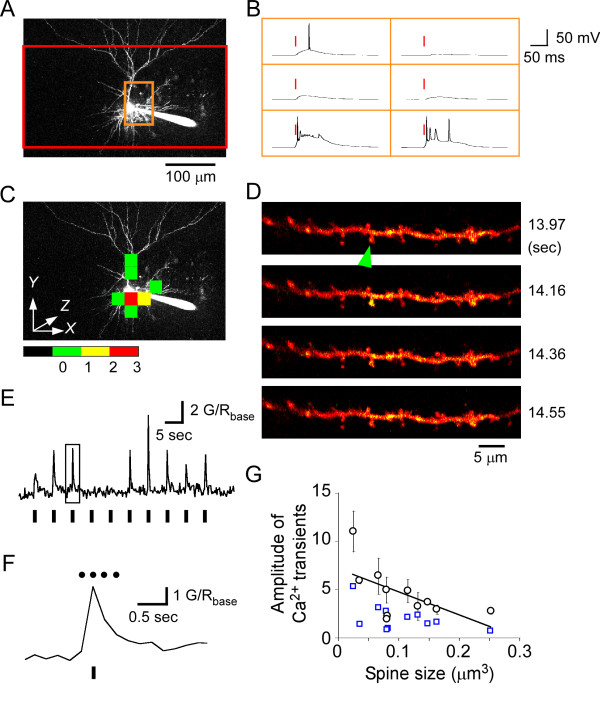
**2pMAPG induction of action potentials in a layer 2/3 cell and detection of Ca^2+ ^transients in a dendritic spine of a layer 5 cell**. (A) A *Z*-stacked image of a representative layer 2/3 pyramidal cell filled with Alexa Fluor 594. The depth of the soma was 82 μm from the slice surface. The red region was divided into 16 × 8 pixels in a single plane and 2pMAPG was performed at each pixel at depths of 30, 80 and 130 μm from the top of the slice. (B) Each trace represents the membrane potential derived from 2pMAPG performed at each pixel in the orange-boxed region in (A) at a depth of 80 μm. Red bars indicate the time of 2pMAPG. (C) Green, yellow, and red pixels indicate AP-evoking pixels detected at any one of the three depths (30, 80 or 130 μm), at any two of these three depths, or at all of the three depths, respectively. (D) A single electrical stimulation near the dendrite induced a Ca^2+ ^transient in one of the dendritic spines in the imaging region (arrow). Red (Alexa Fluor 594) fluorescence and green (Fluo-5F) fluorescence are overlaid. The overlapping signals appear yellow. The time interval between images was 194 ms. The time from the onset of sequential imaging is indicated to the right of each figure. (E) The G intensity/R intensity (G/R) trace in the spine is indicated with an arrow in (D) for 10 electrical stimuli at 0.2 Hz. (F) Expanded G/R trace is boxed in (E). Four dots indicate the acquisition times of the four imaging frames shown in (D). (G) The mean amplitudes of Ca^2+ ^transients (black circle) and the mean 5 coefficients of variance of G/R_base _(blue rectangle) are plotted against spine size. The regression line for Ca^2+ ^transients is shown. Bars, standard error of mean.

**Figure 2:**
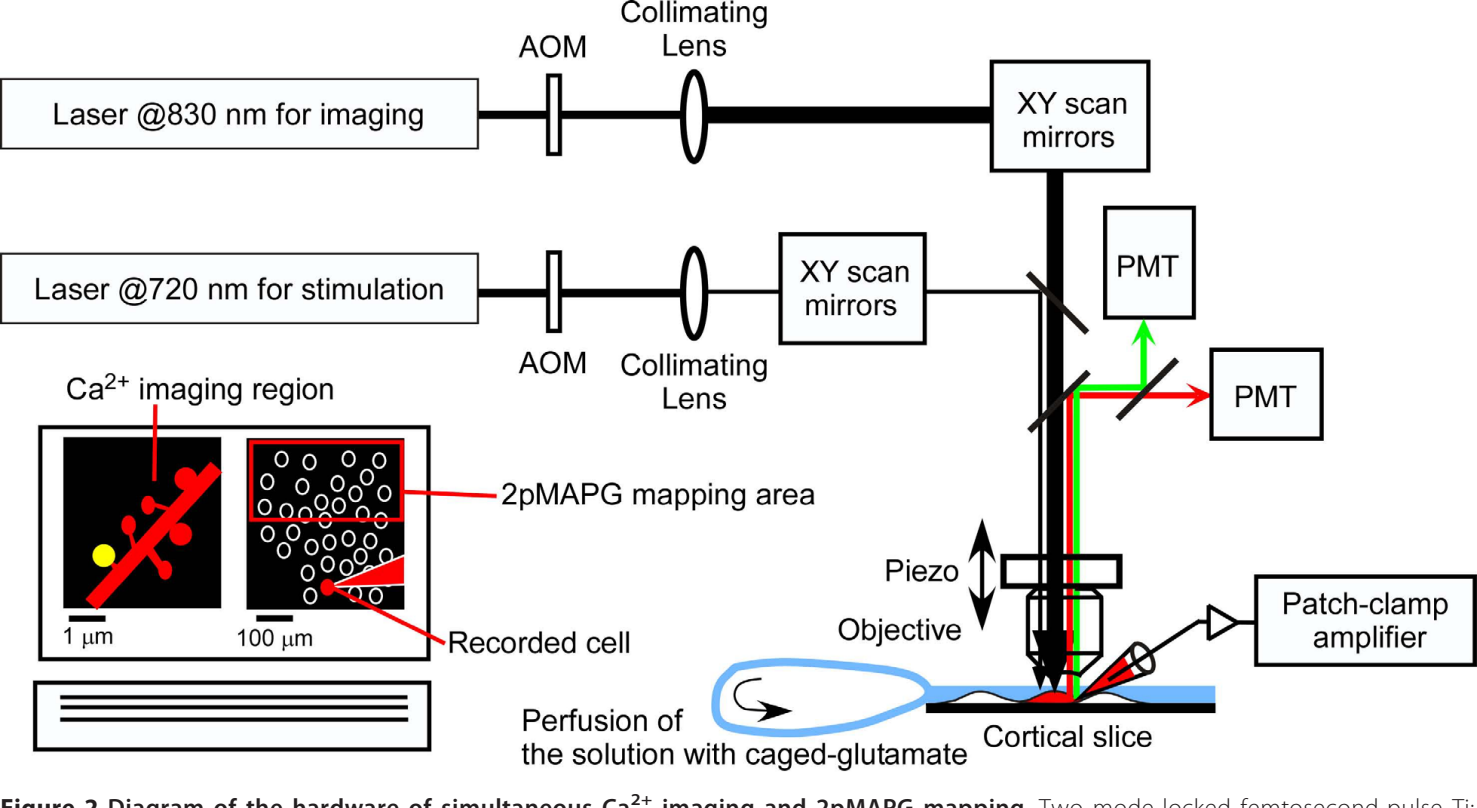
**Diagram of the hardware of simultaneous Ca^2+ ^imaging and 2pMAPG mapping**. Two mode-locked femtosecond-pulse Ti:sapphire lasers set at 830 nm and 720 nm were connected to the laser-scanning microscope via two independent scanheads. The laser beam illumination times and diameters were regulated by acoustic optical modulators (AOMs) and collimating lens, respectively. A pyramidal cell in the cortical slice was patch-clamped and loaded with Alexa Fluor 594 and Fluo-5F. Extracellular solution containing caged glutamate was oxygenated and re-circulated continuously. On the PC screen, the Ca^2+ ^imaging region (left) and 2pMAPG mapping area (right) were chosen. The imaging plane and mapping plane were changed rapidly by the piezo actuator attached to the objective. AOMs, Galvano mirrors, and the piezo actuator were regulated by FV1000-MPE software. Simultaneously, electric signals from the patch-clamp amplifier and from two photomultiplier tubes for detecting red and green fluorescence were recorded. If a photostimulated neuron innervated one of the spines in the imaging region, Ca^2+ ^transients were observed in this spine (yellow in the PC screen). See the details in the main text.

Whole-cell patch clamp recordings were performed on layer 2/3 pyramidal cells from the motor cortex using acute slices, which were perfused with extracellular solution containing caged glutamate (4-carboxymethoxy-5,7-dinitroindolinyl-glutamate, CDNI-glutamate) [20]. The structure of each recorded cell was visualized by loading Alexa Fluor 594 from the whole-cell pipette (Figure 1A) and 2pMAPG was performed at each of 16 × 8 pixels (spacing, 31 μm) within a region of 500 × 250 μm in a single plane. Mapping was carried out at three depths from the slice surface at intervals of 50 μm, so that 2pMAPG occurred at 128 × 3 = 384 points within the 3D area of each recorded cell. The soma of the recorded cell was near the centre of this 3D mapping area. At sites in the perisomatic and proximal dendritic region, 2pMAPG could induce APs, as previously described (Figure 1B and 1C) [7]. This region was supposed to have a larger membrane area included within and/or near the focal spot than that of a thin distal dendritic branch. This difference may cause the total number of activated glutamate receptors to be larger in the former membrane than in the latter, although spine density is lower in the perisomatic area than in the dendritic branch [21]. The observed tendency of perisomatic 2pMAPG to frequently induce APs was similar to that in the case of ultraviolet photostimulation [5]. The number of AP-evoking pixels was 7.9 ± 0.7 per cell (range 5-11, *n *= 8 cells), and the number of APs per AP-evoking pixel was 1.8 ± 0.1 (range 1-4, *n *= 63 pixels). No AP was induced more than 120 ms after the onset of 2pMAPG.

We examined whether the 3D center of the stimulated cell soma could be predicted from the positions of AP-evoking pixels. Comparison of the center of the soma and the average position of the AP-evoking pixels indicated only slight positional differences (-12 ± 12 μm along the *X*-axis, -4 ± 7 μm along the *Y*-axis, and 11 ± 7 μm along the *Z*-axis) using the *XYZ *axes indicated in Figure 1C (*n *= 8 cells). Thus, the average position of AP-evoking pixels predicted the position of the stimulated neuron soma with errors of no more than 12 μm in each direction.

### Identification of dendritic spines innervated by electrically-stimulated axons

Next, electrical stimulation of presynaptic axons was used to examine the reliability of Ca^2+ ^imaging for the detection of Ca^2+ ^transients in dendritic spines. Recoded layer 5 pyramidal cells were filled with Ca^2+ ^indicator (750 μM Fluo-5F; green fluorescence, G) and fluorescent dye (45 μM Alexa Fluor 594; red fluorescence, R). An 830-nm laser beam was used to perform Ca^2+ ^imaging. The Ca^2+ ^imaging regions were approximately 40 × 10 μm in area and included 17 to 31 dendritic spines on each layer 5 pyramidal cell. The acquisition time per imaging frame was 170-320 ms. A stimulation pipette was inserted near the selected dendrite (eight dendritic regions in five cells). Axons adjacent to the dendrite were stimulated 10 to 20 times at 0.2-0.25 Hz during sequential Ca^2+ ^imaging. The membrane potential was held at -30 mV to relieve the Mg^2+ ^block of N-methyl-D-aspartate receptors (NMDARs). The difference between the ratios of G intensity and R intensity (G/R) immediately after and immediately before stimulation, ΔG/R_transient_, was divided by the mean G/R before stimulation (G/R_base_) in the same spine. This ratio was defined as the amplitude of a Ca^2+ ^transient. Ca^2+ ^transients with amplitudes >1 were detected clearly in some spines immediately after a single electrical stimulation of presynaptic axons (Figure 1D-F). Ca^2+ ^transients were not caused by direct electrical stimulation of the observed dendrite, since neither a large inward current with a long decay time nor a widespread rise in Ca^2+ ^transient were observed in the dendritic shaft (Figure 1D). The success rate of Ca^2+ ^transients (1 - failure rate of occurrence of Ca^2+ ^transients) was 0.65 ± 0.08 (*n *= 11 spines from five cells), which probably reflected the reliability of glutamate release. However, as this value might also reflect the failure rate for induction of the axonal AP, it is not discussed here. In successful trials, the amplitude of Ca^2+ ^transients correlated inversely with spine size (Figure 1G; *r *= -0.66, *P *< 0.01, Spearman's rank correlation), consistent with previous results in hippocampal cells [22]. Although ΔG/R_transient _increased with decreasing spine size, the amplitude of Ca^2+ ^transients were >fivefold larger than the coefficient of variance (CV) of G/R_base _in the same spine, regardless of spine size (Figure 1G; *P *< 0.001, paired *t *test). Thus, we concluded that Ca^2+ ^transients could be reliably detected in spines of various sizes under the experimental conditions used here.

### Development of simultaneous 2pMAPG mapping and Ca^2+ ^imaging

**Figure 3:**
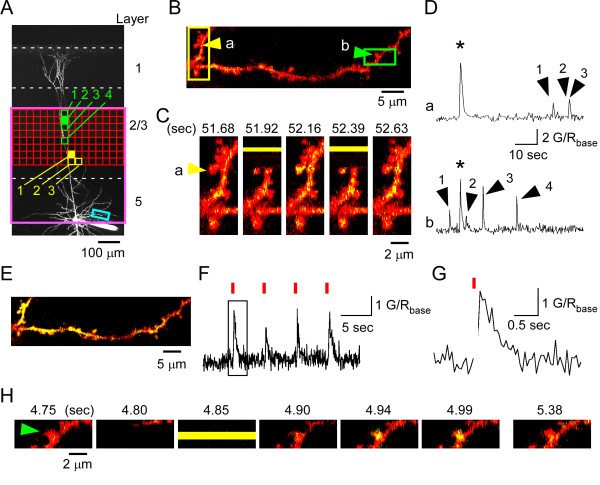
**Induction of Ca^2+ ^transients in the dendritic spines of a layer 5 cell by 2pMAPG mapping in layer 2/3**. (A) A *Z*-stacked image of a representative layer 5 pyramidal cell filled with Alexa Fluor 594. White broken lines indicate laminar borders. The depth of the soma was 53 μm from the slice surface. The red boxes indicate the 2pMAPG mapping area. The sky-blue rectangle indicates the Ca^2+ ^imaging region. The purple box indicates the field of view under the 25× objective lens. (B) Ca^2+ ^imaging region corresponding to the sky-blue rectangle in (A). Red (Alexa Fluor 594) fluorescence and green (Fluo-5F) fluorescence are overlaid. The time interval between images was 236 ms. Dendritic spines indicated by yellow (a) and green (b) arrows exhibited Ca^2+ ^transients after 2pMAPG at the yellow and green pixels in (A), respectively. (C) Five sequential images from the yellow-boxed region in (B). Numbers at the top of the image indicate the onset time of each imaging frame relative to that of the first imaging frame. The yellow horizontal bar in the second frame indicates the time of 2pMAPG at solid yellow pixel 1 in (A). (D) G/R traces in spines shown by the arrows marked a and b in (B) are shown at the top and bottom, respectively. Traces were obtained during 2pMAPG mapping at a depth of 130 μm. Arrows indicate Ca^2+ ^transients immediately after 2pMAPG at the yellow and green pixels in (A). The numbered arrows correspond to the numbered pixels in (A) and the spines shown in (B). Asterisks indicate Ca^2+ ^transients observed throughout the dendrite, possibly due to bursting activity. (E) The image at the time corresponding to the asterisk in (D). (F) The G/R trace in the spine indicated by the green arrow in (B) was obtained by fast imaging of the green-boxed region in (B). The time interval between images was 48 ms. During imaging, 2pMAPG was performed at the green closed pixels in (A) at 0.2 Hz (red bars). (G) Expanded G/R trace boxed in (F). (H) Seven images before, during, and after 2pMAPG in (G).

Next, simultaneous 2pMAPG mapping and Ca^2+ ^imaging were performed by independently scanning 720-nm and 830-nm laser beams (Figure 2). Both the mapping and imaging regions were included in the field of view under a 25× objective lens (purple box in Figure 3A). The 2pMAPG mapping region (500 × 250 μm in a single plane) was located in the lower region of layer 2/3, while the imaging region (~50 × 10 μm) included one or two dendritic branches with 10-40 spines on the recorded pyramidal cell in upper layer 5 (Figure 3A and 3B). In order to perform 2pMAPG mapping at different focal planes from Ca^2+ ^imaging, the focal plane was changed rapidly from the imaging plane to the mapping plane immediately before 2pMAPG at each point. Then, immediately after single 2pMAPG for 9 ms, the focal plane was returned to the imaging plane. The focal plane was moved by regulating a piezo actuator attached to the objective (Figure 2 and Additional File 1: Figure S1A). However, the actuator's rapid movement caused the microscope arm supporting the objective to vibrate for more than 200 ms after the movement, which inhibited stable imaging of the dendritic spines at the target plane (Additional File 1: Figure S1). To absorb the vibration energy and inhibit the vibration, two rubber dampers were attached to the arm and the objective (green arrows in Additional File1: Figure S1A). Damping allowed stabilization of the focal plane at the target plane approximately 60 ms after the onset of axial movement (Additional File 1: Figure S1B-F). The 2pMAPG interval and the frame rate of Ca^2+ ^imaging were set at 470 ms and 150-340 ms, respectively. In this condition, dendritic spines in the imaging plane could be imaged at least once for ~60-400 ms following completion of one 2pMAPG and prior to the start of the next axial movement (Figure 3C and 3H). The mean time from the onset of photostimulation to the peak of the Ca^2+ ^transient was 173 ± 16 ms (*n *= 4 spines). Thus, the Ca^2+ ^transient in a spine was thought to be detected in the first or second imaging frame immediately after 2pMAPG, if the spine was innervated by one of the photostimulated cells. For each mapping plane, complete 2pMAPG at 128 points required ~60 s (128 × 470 ms). Following completion of mapping in one plane, the mapping plane was changed to the next depth, and then 2pMAPG mapping and Ca^2+ ^imaging were again performed simultaneously. Mapping was carried out at 3 depths at intervals of 50 μm.

Reliable measurement of the amplitude or number of excitatory postsynaptic currents (EPSCs) induced by 2pMAPG could not be achieved due to the following experimental side-effects. First, axial movement of the objective caused electric artifacts that continued for >50 ms after the end of 2pMAPG in the whole-cell current measurement (Additional File 1: Figure S1G). Second, since the holding potential was maintained at -30 mV, the driving force of the cation influx was weaker than at -70 mV and the amplitudes of unitary EPSCs were relatively small (<10 pA). Thus, only Ca^2+ ^transients could be considered as synaptic inputs unless otherwise noted.

### Identification of dendritic spines on a layer 5 pyramidal cell innervated by layer 2/3 pyramidal cells

During 2pMAPG mapping, Ca^2+ ^transients were detected in one to three dendritic spines in the first or second imaging frame after 2pMAPG (Figure 3B to 3D). These Ca^2+ ^transients were specific to individual spines and could be distinguished from global Ca^2+ ^increases arising from dendritic spikes possibly triggered by bursting activity (Figure 3E). Following reconstruction of the pixels for 2pMPAG, which were assumed to induce Ca^2+ ^transients, it became apparent that these pixels were frequently attached to each other laterally or axially within the 3D mapping region (yellow pixels in Figure 3A). This finding strongly suggests that one of the neurons associated with these pixels innervated the dendritic spine exhibiting Ca^2+ ^transients, since most AP-evoking pixels (59/63 pixels in eight cells) possessed neighbouring AP-evoking pixels when layer 2/3 pyramidal cells were stimulated by 2pMAPG mapping. However, Ca^2+ ^transients were also detected occasionally in a dendritic spine immediately after 2PMAPG at a given pixel, even though neighboring pixels showed no Ca^2+ ^transient associated with 2pMAPG. These Ca^2+ ^transients might occur spontaneously in a dendritic spine. In order to avoid such contamination, Ca^2+ ^transients were considered to be induced by 2pMAPG when they occurred immediately after 2pMAPGs in at least two neighbouring pixels within a distance of less than 65 μm (approximately the length of two pixels; Additional File 2: Figure S2). These pixels are referred to as grouped Ca^2+ ^transient-evoking pixels (Additional File 2: Figure S2).

For further determination of the reliability of 2pMAPG-induced Ca^2+ ^transients, 2pMAPG was performed repeatedly at one of the grouped Ca^2+ ^transient-evoking pixels with fast Ca^2+ ^imaging (*n *= 4 spines in three cells). Figure 3F-H shows representative Ca^2+ ^transients from one of these spines. The mean success rate in 10 trials was 0.95 ± 0.03 (*n *= 4 spines). If the laser intensity for 2pMAPG was reduced, neither Ca^2+ ^transients nor putative postsynaptic currents were evoked (Additional File 3: Figure S3). These findings indicate that 2pMAPG reliably triggered Ca^2+ ^transients in the spines, and they were not evoked by unrelated, spontaneous glutamate release from presynaptic boutons.

**Figure 4:**
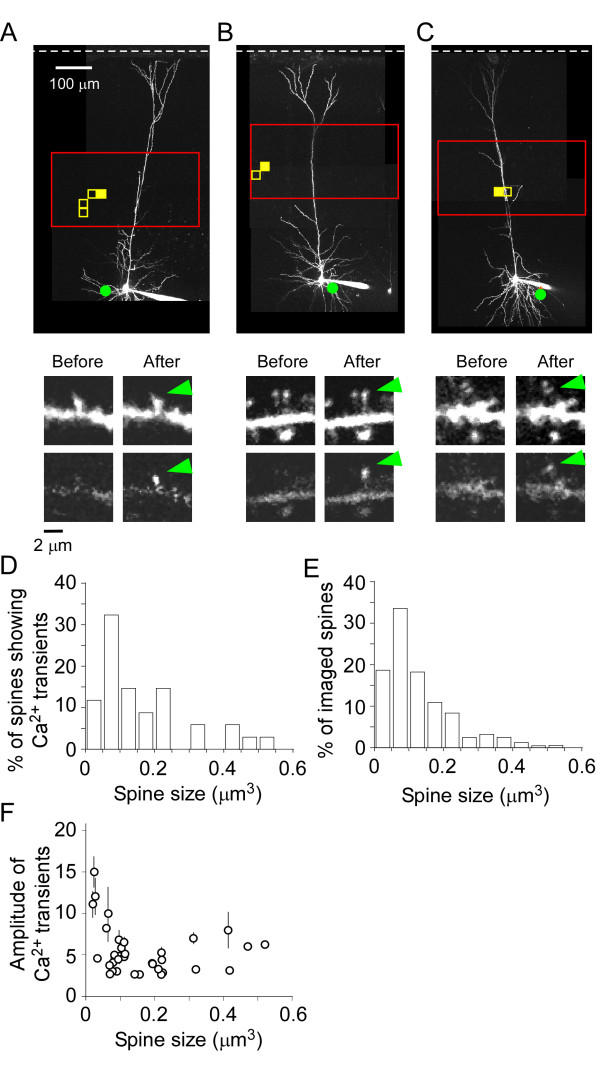
**Spines of various sizes show Ca^2+ ^transients in response to 2pMAPG**. (A-C) Three examples of 2pMAPG mapping and Ca^2+ ^imaging. The top panels show grouped Ca^2+ ^transient-evoking pixels (yellow pixels) and dendritic spines that exhibited Ca^2+ ^transients induced by 2pMAPG (green dots indicate the position of these spines). Red boxes show 2pMAPG mapping areas. White broken lines indicate the pial surface. In the middle and bottom images, green arrows indicate the spines with Ca^2+ ^transients. These images were taken from larger Ca^2+ ^imaging regions. The middle and lower panels show Alexa Fluor 594 and Fluo-5F fluorescence, respectively. The images were taken immediately before and after 2pMAPG at the solid yellow pixels shown in the top panels. (D) The size distribution of spines showing Ca^2+ ^transients in the first or second imaging frame after 2pMAPG (*n *= 34 in 18 cells). (E) The size distribution of all spines included in the imaging regions (*n *= 494 in 18 cells). (F) The mean amplitudes of Ca^2+ ^transients are plotted against spine size. Bars, standard error of mean.

Altogether, Ca^2+ ^transients induced by 2pMAPG at grouped Ca^2+ ^transient-evoking pixels were detected in 34 spines in 25 Ca^2+ ^imaging regions of 18 layer 5 pyramidal neurons. Various sizes of spines exhibited Ca^2+ ^transients (Figure 4A to 4C), and their distribution was similar to that of all the imaged spines (Figure 4D and 4E). The mean and median sizes of all the imaged spines were 0.13 μm^3 ^and 0.10 μm^3^, respectively. The size distribution appeared lognormal, skewing toward large spines, as previously reported in hippocampal pyramidal cells [8,23]. In contrast to the single electrical stimulation (Figure 1G), no inverse relationship between spine size and the amplitude of the Ca^2+ ^transient was detected (Figure 4F; *r *= -0.22, *P *= 0.75, Spearman's rank correlation), possibly because the amplitudes of the Ca^2+ ^transients in four of six large spines (>0.3 μm^3^) were >5. If the reliability of glutamate release tended to be higher with the increase in spine size, as reported in hippocampal synapses, [24,25] each of the multiple APs induced by a single 2pMAPG might consistently release glutamate in the four large spines, resulting in large Ca^2+ ^transients.

### Convergence of synaptic inputs on the same dendritic branch

**Figure 5:**
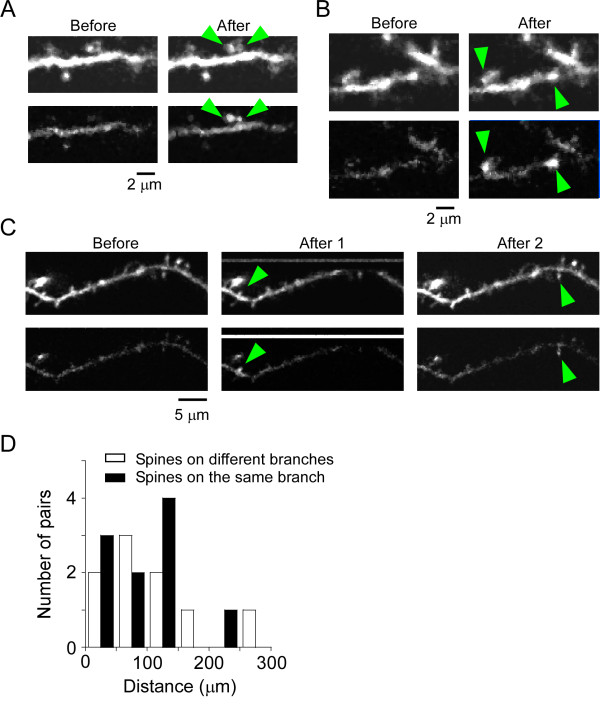
**Convergent projections of layer 2/3 pyramidal cells onto the same dendritic branches**. (A and B) Two pairs of dendritic spines on the same dendritic branches demonstrated simultaneous Ca^2+ ^transients (green arrows). The upper and lower images in each pair show Alexa Fluor 594 and Fluo-5F fluorescence, respectively. The images immediately before and after 2pMAPG at one of the Ca^2+ ^transient-evoking pixels are shown. These images were taken from a larger Ca^2+ ^imaging region. (C) An example of a pair of dendritic spines on the same dendritic branch that showed Ca^2+ ^transients at different times (green arrows). One of the images (Before) was obtained before the start of 2pMAPG mapping and the other two images (After 1 and 2) were taken immediately after 2pMAPG at different pixels. These images were taken from larger Ca^2+ ^imaging regions. (D) Distribution of the distances between somata in pairs of presynaptic cells innervating the same postsynaptic cell. White bars indicate pairs innervating different dendritic branches. Black bars indicate pairs innervating the same dendritic branch.

Ca^2+ ^transients were detected in 1, 2 and 3 dendritic spines from 7, 6 and 5 recorded cells, respectively. The positional relationships of 21 (6 × 1 + 5 × 3) pairs of responding spines were analyzed along with the frequency with which the same presynaptic cell innervated spines on the same dendritic branch. In two instances, 2pMAPG at one group of pixels simultaneously induced Ca^2+ ^transients in a pair of spines in the same imaging region (Figure 5A and 5B). One pair of spines consistently demonstrated Ca^2+ ^transients after stimulation of the same seven pixels in the corresponding mapping region (Figure 5A); in the other pair, Ca^2+ ^transients were induced by 2pMAPG at the same 12 pixels in the corresponding mapping region (Figure 5B). These results indicate that each pair of spines was innervated by the same presynaptic neuron.

In the remaining 19 pairs of dendritic spines, Ca^2+ ^transients were induced by 2pMAPG at different grouped Ca^2+ ^transient-evoking pixels, which suggests that these spines were innervated by different presynaptic neurons. Ten pairs of spines were located on the same dendritic branches, at mean distances of 18 ± 4 μm (2-37 μm). An example is shown in Figure 5C. Nine pairs were located on different dendritic branches of the same postsynaptic cells.

The average position of the grouped Ca^2+ ^transient-evoking pixels was used to predict the position of the presynaptic cells. In order to ascertain whether or not this determination was valid, we reexamined the data for AP induction of layer 2/3 pyramidal cells described in the first part of the Results. Using the same criteria as for the grouped Ca^2+ ^transient-evoking pixels, AP-evoking pixels with neighbouring AP-evoking pixels are referred to as grouped AP-evoking pixels. The differences between the average position of the grouped AP-evoking pixels and the centre of the soma were -14 ± 16 μm along the *X*-axis, 5 ± 8 μm along the *Y*-axis, and 14 ± 7 μm along the *Z*-axis (*n *= 8 cells). Thus, the average position of grouped AP-evoking pixels represented the approximate position of the stimulated neuron soma. The number of grouped AP-evoking pixels per layer 2/3 cell was 7.4 ± 0.5 (*n *= 8 cells), which was more than the number of grouped Ca^2+ ^transient-evoking pixels per spine (5.2 ± 0.5, *n *= 34 spines; *P *< 0.05, Mann-Whitney *U *test). This difference may be due to some AP-evoking pixels with a single AP failing to induce Ca^2+ ^transients in the spine. Assuming that the distribution of grouped Ca^2+ ^transient-evoking pixels was similar to that of grouped AP-evoking pixels, the average position of grouped Ca^2+ ^transient-evoking pixels can be defined as the position of a presynaptic neuron innervating a dendritic spine showing Ca^2+ ^transients.

The distances between the average positions of grouped Ca^2+ ^transient-evoking pixels were calculated. No significant difference in distance from the somata was detected between spine pairs on the same dendritic branch and those on different dendritic branches (Figure 5D; 97 ± 18 μm, *n *= 10 pairs versus 104 ± 27 μm, *n *= 9 pairs, respectively; *P *= 0.87, Mann-Whitney *U *test). In addition, no more than two presynaptic neurons innervating the same dendritic branch within an imaging region could be detected in any 100-μm spherical area. Thus, despite the very small number of dendritic spines showing Ca^2+ ^transients on the same dendritic branch, layer 2/3 presynaptic neurons innervating the same dendritic branch did not appear to be clustered more clearly or frequently than those innervating scattered spines.

### Relationship between the structure and function of dendritic spines on layer 5 pyramidal cells

**Figure 6:**
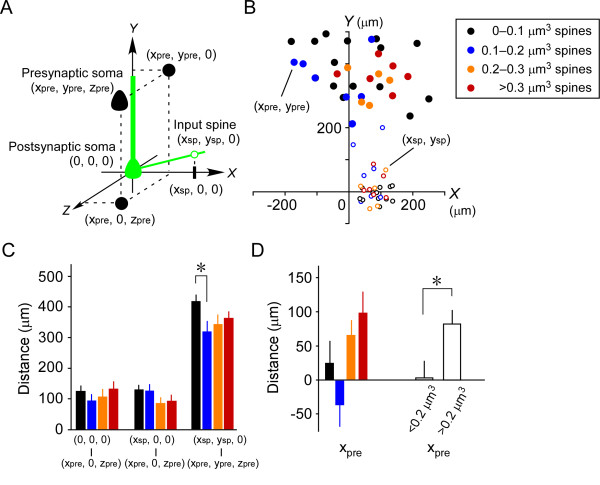
**Relationships between spine size and the location of presynaptic cells**. (A) The soma of the postsynaptic neuron represents the center of the three dimensional coordinates. The presynaptic soma (*x*_pre_, *y*_pre_, *z*_pre_), the innervated spine (*x*_sp_, *y*_sp_, 0), and their projections in the *XY *and *XZ *planes are shown. (B) *XY*-plane-projected distribution of presynaptic neurons (*x*_pre_, *y*_pre_) and the spines they innervated (*x*_sp_, *y*_sp_). Black, blue, orange, and red closed circles represent cells innervating spines of sizes 0-0.1 μm^3 ^(black open circles), 0.1-0.2 μm^3 ^(blue open circles), 0.2-0.3 μm^3 ^(orange open circles), and >0.3 μm^3 ^(red open circles), respectively. (C) Histograms of the lateral distance between the postsynaptic and presynaptic somata (left), $\sqrt{x_{pre}{}^{2}+z_{pre}{}^{2}}$; the lateral distance between the dendritic spine and the presynaptic soma (middle), $\sqrt{{\left(x_{pre}-x_{sp}\right)}^{2}+z_{pre}{}^{2}}$; and the straight-line distance of the dendritic spine from the presynaptic soma (right), $\sqrt{{\left(x_{pre}-x_{sp}\right)}^{2}+{\left(y_{pre}-y_{sp}\right)}^{2}+z_{pre}{}^{2}}$. Black, blue, orange, and red bars indicate spines of sizes 0-0.1 μm^3^, 0.1-0.2 μm^3^, 0.2-0.3 μm^3^, and >0.3 μm^3^, respectively. *P < 0.05 (one-way ANOVA followed by Tukey's test). (D) Histograms of *x*_pre_. Black, blue, orange, and red bars indicate the presynaptic cells innervating the spines of sizes 0-0.1 μm^3^, 0.1-0.2 μm^3^, 0.2-0.3 μm^3 ^and >0.3 μm^3^, respectively. * *P *< 0.05 (Mann-Whitney *U *test).

New 3D coordinates were defined to clarify the relationships between the size and location of innervated dendritic spines and the locations of pre- and postsynaptic neuronal somata (Figure 6A). (1) The postsynaptic neuronal soma is set at the centre of the coordinates (0, 0, 0). (2) The main apical dendrite of the postsynaptic neuron is assumed to be in a line that follows the positive *Y *axis. (3) The location of each identified spine is aligned at (*x*_sp_, *y*_sp_, 0) in the *XY *plane with *x*_sp _> 0. Thus, *x*_sp _indicates the lateral distance between the dendritic spine and the soma. (4) In this 3D coordinate system, (*x*_pre_, *y*_pre_, *z*_pre_) is defined as the position of the presynaptic neuron innervating the dendritic spine showing Ca^2+ ^transients, as described above.

First, we examined the relationships between spine size and each of the positional parameters. Spine size did not correlate with *x*_sp_, which varied from 20 to 140 μm (*r *= 0.05, *n *= 34, *P *= 0.77; Spearman's rank coefficient). Spines were classified into four groups according to size (Figure 6B; spines of 0-0.1 μm^3^, *n *= 15; 0.1-0.2 μm^3^, *n *= 8; 0.2-0.3 μm^3^, *n *= 5 and >0.3 μm^3^, *n *= 6). The lateral distance between the postsynaptic and presynaptic somata $\sqrt{x_{pre}{}^{2}+z_{pre}{}^{2}}$, and the lateral distance between the dendritic spine and the presynaptic soma $\sqrt{{\left(x_{pre}-x_{sp}\right)}^{2}+z_{pre}{}^{2}}$, were not significantly different across the four groups (Figure 6C; *P *= 0.68 and 0.39, respectively, one-way ANOVA). The straight-line distance of the dendritic spine from the presynaptic soma $\sqrt{{\left(x_{pre}-x_{sp}\right)}^{2}+{\left(y_{pre}-y_{sp}\right)}^{2}+z_{pre}{}^{2}}$, was significantly longer in spines of 0-0.1 μm^3 ^than in those of 0.1-0.2 μm^3 ^(Figure 6C; *P *< 0.05, one-way ANOVA followed by Tukey's test). Presynaptic cells innervating spines of >0.2 μm^3 ^were preferentially distributed in the field ipsilateral to the spine (nine of 11 cells; orange and red closed circles in Figure 6B). In fact, the *x*_pre _of spines >0.2 μm^3 ^was significantly greater than that of spines <0.2 μm^3 ^(Figure 6D; 83 ± 20 μm versus 3 ± 25 μm, respectively; *P *< 0.05, Mann-Whitney *U *test). However, no significant differences in *x*_pre _were detected between the four size groups (Figure 6D; *P *= 0.11, one-way ANOVA).

**Figure 7:**
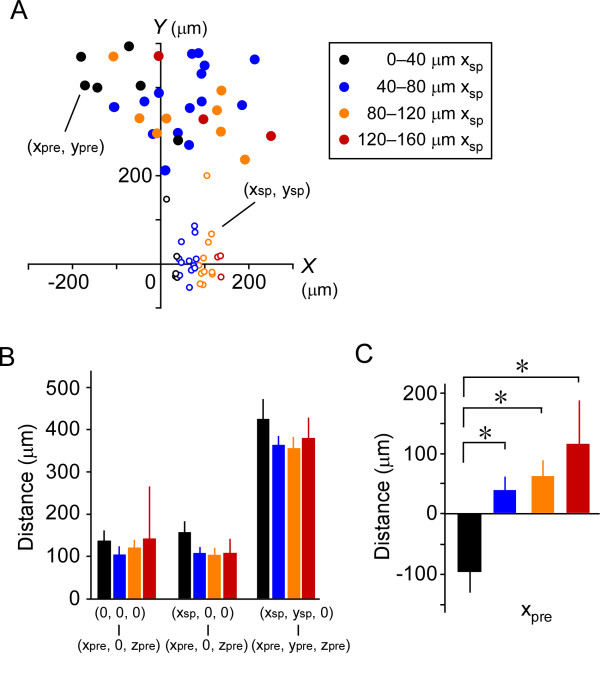
**Relationships between spine location and the location of the presynaptic cells**. (A) *XY*-plane-projected distribution of presynaptic neurons (*x*_pre_, *y*_pre_) and the spines they innervated (*x*_sp_, *y*_sp_). Black, blue, orange and red closed circles represent the cells innervating spines with an *x*_sp _of 0-40 μm (black open circles), 40-80 μm (blue open circles), 80-120 μm (orange open circles), and >120 μm (red open circles), respectively. (B) Histograms of the lateral distance between the postsynaptic and presynaptic somata (left), $\sqrt{x_{pre}{}^{2}+z_{pre}{}^{2}}$; the lateral distance between the dendritic spine and the presynaptic soma (middle), $\sqrt{{\left(x_{pre}-x_{sp}\right)}^{2}+z_{pre}{}^{2}}$; and the straight-line distance of the dendritic spine from the presynaptic soma (right), $\sqrt{{\left(x_{pre}-x_{sp}\right)}^{2}+{\left(y_{pre}-y_{sp}\right)}^{2}+z_{pre}{}^{2}}$. Black, blue, orange and red bars indicate spines with an x_sp _of 0-40 μm, 40-80 μm, 80-120 μm, and 120-160 μm, respectively. (C) Histograms of *x*_pre_. Black, blue orange and red bars indicate the presynaptic cells innervating the spines with an *x*_sp _of 0-40 μm, 40-80 μm, 80-120 μm and 120-160 μm, respectively. *P < 0.05 (one-way ANOVA followed by Tukey's test).

We next examined the relationships between spine location (*x*_sp_) and each of the other positional parameters. Spines were classified into four groups according to *x*_sp _(Figure 7A; spines with an *x*_sp _of 0-40 μm, *n *= 6; 40-80 μm, *n *= 14; 80-120 μm, *n *= 11; and >120 μm, *n *= 3). No significant difference in the lateral distance of the postsynaptic soma from the presynaptic soma, the lateral distance of the dendritic spine from the presynaptic soma, or the straight-line distance of the dendritic spine from the presynaptic soma was detected across groups (Figure 7B; *P *= 0.81, 0.32 and 0.51, respectively, one-way ANOVA). However, *x*_pre _in the 0-40-μm group was significantly different from the other groups (Figure 7C; *P *< 0.05, one-way ANOVA followed by Tukey's test). This difference was supported by the fact that *x*_pre _correlated with *x*_sp _(*r *= 0.46, *P *< 0.01, Spearman's rank coefficient). It is unlikely that this difference was due to the cutting of axon fibers during the slice preparation, since *y*_pre _did not correlate with *y*_sp _(*r *= 0.25, *P *=0.15, Spearman's rank coefficient). These results suggest that more laterally located dendritic spines received synaptic inputs from laterally located cells in the field ipsilateral to the spine, independent of the lateral distance between the presynaptic soma and the dendritic spine.

## Discussion

This study developed a novel method for simultaneously performing Ca^2+ ^imaging in a narrow region that includes a segment of a dendrite, and 2pMAPG across a broad area that includes many pyramidal cells. The methodological focus was the simultaneous use of two two-photon lasers that over- and under-filled the back aperture of the objective for Ca^2+ ^imaging of dendritic spines and photostimulation of neurons, respectively. It was important to inhibit vibration of the objective, which accompanied the rapid axial movement between the imaging plane and the photostimulation plane. This inhibition was achieved by attaching dampers to the objective. This novel method allowed identification of spines innervated by some of the systematically stimulated neurons over a relatively broad 3D area.

In contrast to a paired recording where the presynaptic cell is identified precisely, this method required prediction of the location of the presynaptic cell from the grouped Ca^2+ ^transient-evoking pixels. The Ca^2+ ^transient might be generated by a directly photostimulated presynaptic cell or by a presynaptic cell synaptically activated by multiple photostimulated neurons. To clarify this issue, we estimated the number of AP-evoking cells per 2pMAPG (N_APcell_). According to our previous report, [7] N_APcell _is estimated as N_APpixel _× V_pixel _× ρ_cell_, where N_APpixel _is the number of AP-evoking pixels per cell during 2pMAPG mapping, V_pixel _is the volume of a single pixel for 2pMAPG, and ρ_cell _is the density of excitatory pyramidal neurons. Assuming that N_APpixel_, V_pixel_, and ρ_cell _in layer 2/3 were 7.9, 31 × 31 × 50 μm, and 6.8 × 10^-5 ^μm^-3 ^[7], respectively, the N_APcell _in layer 2/3 was 26.

According to Holmgren *et al*., [26] the connection probability between nearby (distance of <25 μm) layer 2/3 pyramidal neurons is 0.18, and the average amplitude of excitatory post-synaptic potentials (EPSPs) between connected pairs of neurons is 0.65 mV. Thus, if the APs induced in 26 nearby cells simultaneously depolarize a postsynaptic cell, its depolarization is estimated at 26 × 0.18 × 0.65 = 3 mV. Even if each of the stimulated cells generated three APs simultaneously, the total synaptic depolarization will be 9 mV, which is not large enough to generate an AP in the postsynaptic cell. In fact, at any 2pMAPG pixel that was not associated with the soma or proximal dendrites of the recorded cell, no AP was induced in a recorded layer 2/3 cell during 2pMAPG mapping. However, if 2pMAPG directly depolarized a cell near the stimulated pixel and its depolarization did not reach the threshold for generation of an AP, synaptic inputs from its nearby photostimulated cells might assist the cell to generate an AP. If so, such pixels should have been involved in grouped AP-evoking pixels during the mapping of AP induction, which were used for the prediction of neuronal position. Thus, the approximate position of the presynaptic cell could be determined from the positions of the grouped Ca^2+^-evoking pixels, although the exact cell cannot be identified.

We found that the distribution of innervating neurons in the local neocortical circuits appears to depend upon the lateral distance of the dendrite from the soma. This finding does not contradict the results of anatomical studies in which overlapping axonal and dendritic arbors were reconstructed [16,17,27-30]. Axons and dendrites tend to extend isotropically from the soma, and the density of their arbors decreases laterally over a few hundred microns. Thus, if presynaptic neurons are located on one side of the postsynaptic cell, the presynaptic axons would overlap with dendrites on the same side of the postsynaptic cell more frequently than those on the other side. We also found that large spines (>0.2 μm^3^) tended to be innervated by cells on the ipsilateral side, while small spines (<0.2 μm^3^) tended to be innervated by cells on both sides. These findings suggest that each dendritic branch may have a different receptive field in which the innervating neurons are located, and that this receptive field may be mediated primarily by large spines. However, it should be noted that synapses with a very low release probability or very low expression of NMDAR may be underestimated under the conditions used in this study.

It can be assumed theoretically that a functionally-associated group might innervate a specific dendritic branch of the postsynaptic cell to generate a nonlinear summation of synaptic inputs in the local dendrite [31-33]. In some invertebrates and vertebrates, distinct sensory inputs induce Ca^2+ ^signals in specific dendrites in the visual and auditory systems [13,34]. In the mammalian neocortex, functionally-associated cells are assumed to connect with each other with a higher probability, forming microcircuits [35-37]. However, these cells are not necessarily neighbours and, in this study, no clear clustering of presynaptic cells innervating the same dendritic branch was observed. Candidates for functionally connected and associated groups include subsets of neurons derived from the same stem cell [38] and subsets of the neurons innervating the same brain area [19,39]. These subsets of neurons can be visualized specifically, and 2pMAPG can be used to stimulate visually-identified neurons at the level of a single cell [6]. Thus, the detection method described here will allow clarification of whether or not such groups of neurons innervate the same dendritic branch, what part of dendrites they innervate, and what size of spines they innervate.

Two limitations to the present method need to be overcome in the future. First, the present rate of Ca^2+ ^imaging is not fast enough to observe more than ~100 spines at once. In this study, the maximum number of spines that could be imaged simultaneously was 40, which represents only a few percent of the total number of spines on a single pyramidal cell. In order to observe more than a hundred scattered dendritic spines, 3D arbitrary movement of the scanning points will be useful [40]. Second, this study was performed using slice preparations and, inevitably, axon fibres crossing the cutting plane were severed. The depth of the mapping area was also limited by slice thickness. Thus, it was difficult to clarify the distribution of all the presynaptic neurons innervating all the spines in the imaging region. Ideally, the mapping and imaging should be performed *in vivo*. *In vivo *Ca^2+ ^imaging of dendritic spines has been reported, whereas *in vivo *two-photon uncaging of glutamate has not. However, 2pMAPG will be more applicable to *in vivo *studies than ultraviolet photostimulation, since the infrared (720-nm) light required for 2pMAPG can penetrate deeper into brain tissue than ultraviolet light with one-photon excitation. It has been reported recently that a newly-developed caged glutamate, RuBi-glutamate, can be activated by two-photon excitation at the longer wavelength of 800 nm [41]. Alternatively, two-photon stimulation of Channelrhodopsin-2 (ChR2), a light-gated cation channel, might be more applicable to *in vivo *stimulation, since ChR2 excitation does not require the perfusion of any exogenous agent within the brain. In addition, the longer wavelength used for ChR2 excitation (920 nm) will stimulate a deeper cortical area than can be achieved with either CDNI- or RuBi-glutamate. As a large excitation volume is essential for two-photon excitation of ChR2 (MM unpublished data) [42], two-photon macro stimulation will be effective. Development of novel caged compounds and ChR2 variants will improve the performance of two-photon stimulation of neurons *in vivo*. In the future, specific clusters or specific distributions of presynaptic cells that could not be detected in this study may be revealed by combining photostimulation and fast Ca^2+ ^imaging of more than 100 spines *in vivo*.

## Conclusions

This study developed a novel method for simultaneously performing Ca^2+ ^imaging in a narrow region that includes a segment of a dendrite and 2pMAPG across a broad area that includes many pyramidal cells. This technique allowed identification of various sizes of innervated spine located <140 μm laterally from the postsynaptic soma. Spines distal to their parent soma were preferentially innervated by cells on the ipsilateral side. Large spines (>0.2 mm^3^) tended to be innervated by cells on the ipsilateral side, while small spines (<0.2 mm^3^) tended to be innervated by cells on both sides. However, no cluster of neurons innervating the same dendritic branch was detected. The method described here is a valuable first step toward elucidating the basic microarchitecture of connectivity between neurons and synapses, and the stimulation of presynaptic cells that may induce nonlinear dendritic integration in the postsynaptic cell.

## Methods

### Slice preparation

Slices (350-μm thick) of motor cortex were prepared from 17- to 20-day-old Sprague-Dawley rats in accordance with the procedure described by Kawaguchi et al., [43] using a cutting solution containing 120 mM choline chloride, 3 mM KCl, 8 mM MgCl_2_, 1.25 mM NaH_2_PO_4_, 26 mM NaHCO_3 _and 25 mM glucose. Slices were incubated at 32°C for 30 min and then stored in an incubation chamber at room temperature (22°-25°C) for at least 1 h. Each slice was transferred to a recording chamber at room temperature. The extracellular solution contained 125 mM NaCl, 2.5 mM KCl, 2-3 mM CaCl_2_, 1 mM MgCl_2_, 1.25 mM NaH_2_PO_4_, 26 mM NaHCO_3_, 20 mM glucose, 200 μM Trolox (Aldrich, WI, USA), and 1.5 mM CDNI-glutamate [20]. The extracellular solution (2-4 mL) was oxygenated and recirculated continuously. All experiments were approved by the animal experimentation committee of the Faculty of Medicine, University of Tokyo.

### Electrophysiology

Patch-clamp electrodes (open-tip resistance, 4-8 MΩ) were filled with a solution containing 135 mM cesium gluconate, 4 mM MgCl_2_, 10 mM disodium phosphocreatine, 4 mM Na-ATP, 0.4 mM Na-GTP, 10 mM HEPES-CsOH, 45 μM Alexa Fluor 594 and 0.75 mM Fluo-5F (pH 7.2, 293 mOsm). QX314 (5 mM) and D600 (0.5 mM) were also included to minimize the occurrence of dendritic spikes and Ca^2+ ^influx via voltage-gated Ca^2+ ^channels. In Ca^2+ ^imaging of dendritic spines during 2pMAPG mapping, recordings were obtained from layer 5 pyramidal cells in the agranular area located 60 ± 13 μm [mean ± standard deviation (SD), *n *= 18] deep and 825 ± 65 (SD) from the pia. Series resistance was 17 ± 7 (SD) MΩ. The Ca^2+ ^imaging regions were 47 ± 23 (SD) μm deep (*n *= 25). During Ca^2+ ^imaging, the membrane potential was held at -30 mV to remove the Mg^2+ ^block of NMDARs. The liquid junction potential was not corrected. In experiments to detect APs in layer 2/3 pyramidal neurons under the whole-cell current-clamp mode, the intracellular solution contained 138 mM potassium gluconate, 4 mM MgCl_2_, 10 mM disodium phosphocreatine, 50 μM Alexa Fluor 594, 4 mM Na-ATP, 0.3 mM Na-GTP and 10 mM HEPES-KOH (pH 7.2, 297 mOsm). The mean resting potential of the cells was -72 ± 3 (SD) mV (*n *= 8). Data were low-pass filtered at 2 kHz, sampled at 5-10 kHz, and recorded using FV1000-MPE software (Olympus, Tokyo, Japan).

Electrical stimulation was performed using a glass pipette filled with Alexa Fluor 594 dissolved in extracellular solution containing 2 mM Ca^2+^. Alexa Fluor 594 was used to visualize the position of the pipette and to keep it away from the dendrite. A current of 20-50 μA was applied for 0.1 ms per stimulation.

### Two-photon excitation imaging and uncaging of glutamate

Experiments were performed using an upright microscope (BX61WI; Olympus) and an FV1000-MPE laser-scanning microscope system. Since Ca^2+ ^imaging required high spatial resolution and 2pMAPG mapping should be performed over a broad area, a water-immersion objective with a high-numerical-aperture (NA) and low-magnification configuration (XLUMPlanFI/IR 25×, NA of 1.05) was used. Two mode-locked femtosecond-pulse Ti:sapphire lasers (MaiTai HP and MaiTai HP DeepSee; Spectra Physics, CA, USA) set at 720 and 830 nm were connected to the laser-scanning microscope via two independent scanheads (Figure 2). The laser emitted from the MaiTai HP was chirp compensated prior to entering the scanhead.

For 2pMAPG, the diameter of the 720-nm laser beam was adjusted to underfill the back aperture of the objective. This adjustment was achieved by using a motor-driven stage (SGSP20-85; Sigma-Koki, Tokyo, Japan) to change the distance between the two convex lenses in the optical pathway prior to entering the scanhead (Figure 2). As a result, the effective NA was small and the focal volume was large. In addition, the laser intensity was increased to release much more caged glutamate and, therefore, to activate many more glutamate receptors near the focal volume than could be achieved at the diffraction limit [7]. This modification allowed effective induction of APs in cells near the focal volume.

In order to image dendritic spines at high resolution, the diameter of the 830-nm laser beam was adjusted to overfill the back aperture of the objective. The FWHM of the focal volume of the laser beam at 830 nm was estimated to be 0.41 ± 0.01 (SEM) μm laterally and 1.57 ± 0.02 (SEM) μm axially (*n *= 10). The illumination time of the lasers was regulated by acoustic optical modulators (Figure 2). Fluorescence emitted from the specimen was separated using a 560-nm dichroic mirror (FF560; Semrock, NY, USA) and one of two barrier filters (FF01-510/84 [Semrock] or HQ 620/60 [Chroma Technology, VT, USA]), followed by detection with photomultiplier tubes in the green (G) and red (R) fluorescence, respectively (Figure 2).

Images of neuronal structure were acquired by two-dimensional scanning with the 830-nm laser at different depths and these images were stacked perpendicular to the image plane. Pixel lengths for imaging whole neurons and dendritic spines were 0.96 μm and 0.08-0.16 μm, respectively. In all figures with fluorescent images of whole neurons, the top of the image is closest to the pial surface. The locations of recorded cells and laminar borders were identified under trans-illumination of the 830-nm laser scanning with a low-magnification objective (MPlan N 5x, NA of 0.1).

For 2pMAPG mapping, 128 pixels (16 × 8, spaced 31 μm apart) were scanned. Within each pixel, laser-mediated photolysis at 720 nm was performed consecutively at 3 × 3 points using lateral intervals of 6 μm with a pulse-train duration of 9 ms (1 ms at each point) [7]. The mapping was performed at 3 different depths, each separated by 50 μm. Brain tissue scatters light, and the strength of scattering is described by the average length of the distance between scattering events (l_s_) [44]. To maintain constant laser power for photolysis (P = 32-35 mW) within the mapped plane at depth *z *from the slice surface, the laser power was adjusted to P/e^-z/ls ^before entering the tissue slice, with l_s _set to 80 μm. The focal plane was moved by regulating a piezo actuator (PI 721, Physik Instrumente, Karlsruhe, Germany) attached to the objective (Figure 2 and Additional File 1: Figure S1A). In contrast to our previous experiments of 2pMAPG mapping to detect EPSCs, [7] Ca^2+ ^imaging required a long time interval between each 2pMAPG (470 ms versus 100 ms) because Ca^2+ ^transient decay (>200 ms) was much slower than EPSC decay (~20 ms). In addition, photobleaching of the fluorescence depended on the duration of imaging. Thus, the space between neighbouring 2pMAPG pixels was increased from 19 μm to 31 μm laterally and from 25-30 μm to 50 μm axially and the total number of 2pMAPG mapping pixels was reduced from 3072-5120 to 384. In order to keep the number of AP-evoking pixels during this rough mapping similar to that in our prior report (~8), the focal volume for 2pMAPG was increased approximately three-fold from the previous experiments [7].

### Data analysis

Spine size was estimated as described previously [22] FWHM was measured for the heads of large, sphere-like spines (criterial spines) and then fitted to the FHWM-diameter curve, followed by estimation of the diameter and volume of the head. [22]. After that, the volumes of other spines were estimated based on total fluorescence intensity. Ca^2+ ^transients that occurred in spines during the first or second imaging frame after stimulation and with amplitude larger than the mean of G/R_base _and 5 CV of G/R_base _were analyzed as described in the main text. FV1000-MPE software, IPLab (BD Biosciences, MD, USA) and our own software programs based on LabView (National Instruments, TX, USA) were used for image processing. Data are presented as means ± SEM, unless stated otherwise. Error bars on graphs correspond to the SEM. Mann-Whitney *U *test, Spearman's rank correlation, Student's paired *t *test, and one-way ANOVA were used for statistical comparisons. A *P *value of < 0.05 was used as the criterion for a significant statistical difference.

## Competing interests

The authors declare that they have no competing interests.

## Authors' contributions

MM conceived of the study, carried out the experiments, analyzed the data, and drafted the manuscript. GCRE-D synthesized CDNI-glutamate and helped to draft the manuscript. YK helped to perform the experiments. HK participated in the design of the study, provided resources and support for the experiments and helped to draft the manuscript. All authors read and approved the final manuscript.

## Supplementary Material

Additional file 1 **Rapid axial movement driven by a piezo actuator**. The focal plane was moved by regulating a piezo actuator attached to the objective with dampers. Click here for file

Additional file 2 **Grouped Ca^2+ ^transient-evoking pixels**. If Ca^2+ ^transients in a spine occurred in the imaging frames immediately after 2pMAPG at the green pixel and at least one of the red pixels, these pixels were referred to as grouped Ca^2+ ^transient-evoking pixels. Click here for file

Additional file 3 **Correlation between the occurrence of Ca^2+ ^transients and putative postsynaptic currents**. If the laser intensity for 2pMAPG was reduced, neither Ca^2+ ^transients nor putative postsynaptic currents were evoked. Click here for file
