# Supplementary material for: Simultaneous two-photon activation of presynaptic cells and calcium imaging in postsynaptic dendritic spines
Source: Neural Syst Circuits. 2011 Jan 26;1:2. doi: 10.1186/2042-1001-1-2 (PMC3269225; doi:10.1186/2042-1001-1-2)
Supplement: Additional file 1 — Rapid axial movement driven by a piezo actuator. The focal plane was moved by regulating a piezo actuator attached to the objective with dampers. [file 2042-1001-1-2-S1.PDF]

Additional file 1

Figure S1

Rapid axial movement driven by a piezo actuator

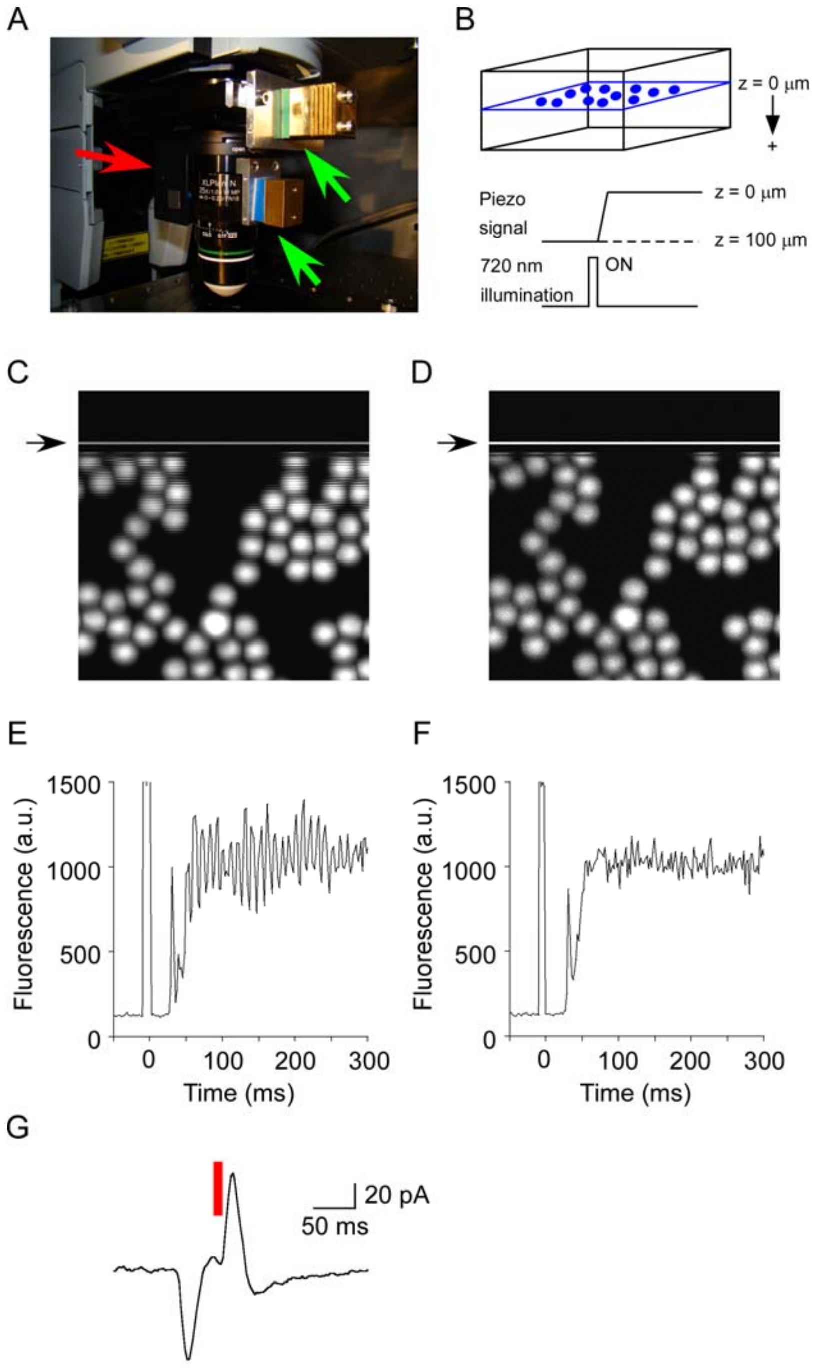

(A) Image of the objective, piezo actuator (red arrow), and two dampers (green arrows). (B) Experimental scheme for the images shown in (C–F). Fluorescent beads (3 μm) were placed on a cover glass at z = 0 μm. The 2-dimensional (C and D) and fast line (E and F) scans were performed using the 830-nm laser. The 720-nm laser was illuminated for 10 ms, 190 ms after onset of the scan with the 830-nm laser. At the end of the 720-nm illumination, the focal plane of the objective was changed rapidly from 100 μm to 0 μm by an electric signal from the piezo driver. (C) Image quality without the dampers. After illumination with the laser at 720 nm (arrow), the image of the beads showed many stripes due to vibration of the objective along the optical path. The time per imaging frame was 1.1 s. (D) Image quality with the dampers. The repeated stripes were not observed. (E) Representative time course of fluorescence intensity of a bead taken by line scanning without dampers. Due to vibration of the objective, the fluorescence intensity at z = 0 μm did not stabilize for more than 200 ms after the end of the illumination with the 720-nm laser (time 0). (F) Representative time course of the fluorescence intensity of a bead taken by line scanning with dampers. The dampers absorbed the vibrational energy, and the imaging plane stabilized at the target plane (z = 0 μm) approximately 60 ms after the end of the illumination with the 720-nm laser. (G) Representative trace of the whole-cell current when axial movement occurred. The axial distance between the 2pMAPG mapping and Ca^{2+} imaging planes was 58 μm. The red bar indicates the illumination time (9 ms) of the laser at 720 nm. The electric command driving the movement from the imaging plane to the mapping plane was initiated 61 ms before the onset of stimulation. The electric command driving the movement

from the mapping plane to the imaging plane was initiated immediately after the end of stimulation.
