# Supplementary material for: Simultaneous two-photon activation of presynaptic cells and calcium imaging in postsynaptic dendritic spines
Source: Neural Syst Circuits. 2011 Jan 26;1:2. doi: 10.1186/2042-1001-1-2 (PMC3269225; doi:10.1186/2042-1001-1-2)
Supplement: Additional file 2 — Grouped Ca2+ transient-evoking pixels. If Ca2+ transients in a spine occurred in the imaging frames immediately after 2pMAPG at the green pixel and at least one of the red pixels, these pixels were referred to as grouped Ca2+ transient-evoking pixels. [file 2042-1001-1-2-S2.PDF]

Additional file 2

Figure S2

Grouped Ca^{2+} transient-evoking pixels

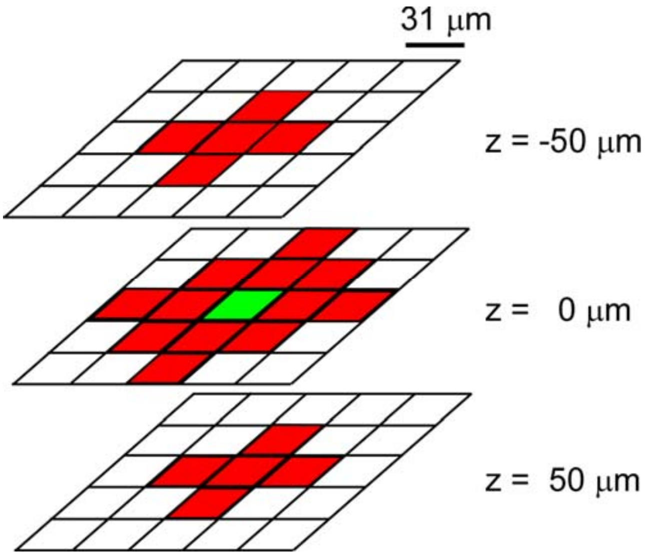

If Ca^{2+} transients in a spine occurred in the imaging frames immediately after 2pMAPG at the green pixel and at least one of the red pixels, these pixels were referred to as grouped Ca^{2+} transient-evoking pixels.
