# Supplementary material for: Simultaneous two-photon activation of presynaptic cells and calcium imaging in postsynaptic dendritic spines
Source: Neural Syst Circuits. 2011 Jan 26;1:2. doi: 10.1186/2042-1001-1-2 (PMC3269225; doi:10.1186/2042-1001-1-2)
Supplement: Additional file 3 — Correlation between the occurrence of Ca2+ transients and putative postsynaptic currents. If the laser intensity for 2pMAPG was reduced, neither Ca2+ transients nor putative postsynaptic currents were evoked. [file 2042-1001-1-2-S3.PDF]

Additional file 3

Figure S3

Correlation between the occurrence of Ca^{2+} transients and putative postsynaptic currents

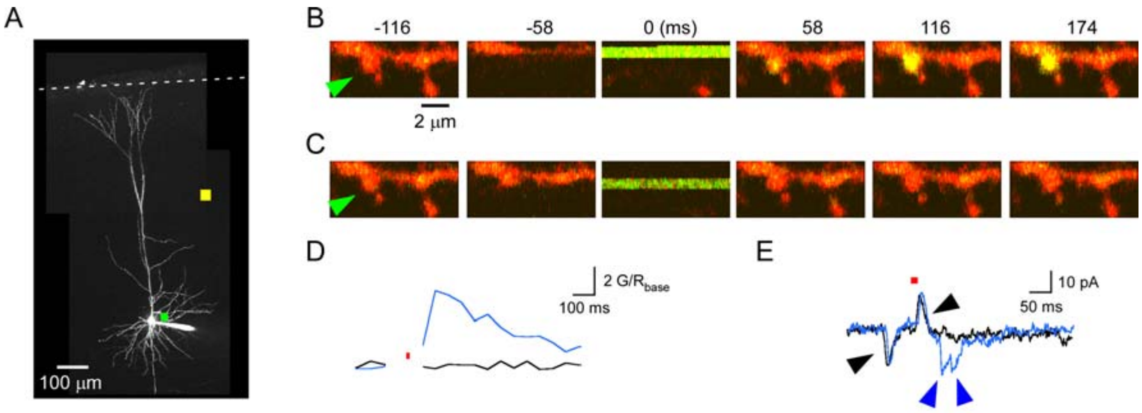

(A) A stacked image of a representative layer 5 pyramidal cell filled with Alexa Fluor 594. The white broken line indicates the pial surface. The depth of the soma was 58 μm from the slice surface. The green dot indicates the location of a spine that showed Ca^{2+} transients after 2pMAPG at the yellow pixel; the depth of the yellow pixel from the slice surface was 80 μm. (B) The location of the six sequential images including the spine is indicated by the green dot in (A). Red (Alexa Fluor 594) fluorescence and green (Fluo-5F) fluorescence are overlaid. The overlapping signals appear yellow. The intensity of the 720-nm laser was 84 mW. Time per imaging frame was 58 ms. The times listed above the images are their acquisition times. The image corresponding to 2pMAPG (the third panel) was set to 0 ms. (C) When the intensity of the 720-nm laser was 42 mW, no Ca^{2+}

transient was induced in the spine indicated by the green arrow. Experiments using the laser at different powers were performed twice, and the results were similar. (D) G/R traces in the spine indicated by the arrows in (B) and (C) when 2pMAPG was performed. Blue and black traces correspond to (B) and (C), respectively. The red bar indicates the time of 2pMAPG. (E) The traces of whole-cell currents corresponding to (D). The holding potential was -30 mV. Blue arrows indicate putative postsynaptic currents induced by 2pMAPG at a laser intensity of 84 mW. These currents were not induced when the laser intensity was 42 mW. Black arrows indicate artifacts caused by axial movement of the objective (see Additional file 1: Fig. S1G). The axial distance between the imaging and 2pMAPG planes was 8 μm.
